# Multi-targeted gene silencing strategies inhibit replication of *Canine morbillivirus*

**DOI:** 10.1186/s12917-020-02671-2

**Published:** 2020-11-19

**Authors:** Otávio Valério de Carvalho, Marcus Rebouças Santos, Juliana Lopes Rangel Fietto, Mauro Pires Moraes, Márcia Rogéria de Almeida, Gustavo Costa Bressan, Lindomar José Pena, Abelardo Silva-Júnior

**Affiliations:** 1 Laboratory of Immunobiological and Animal Virology, Department of Veterinary Medicine, Federal University of Viçosa, Av. Peter Henry Rolfs, s/n, Viçosa, MG 36570-000 Brazil; 2 Department of Virology and Experimental Therapy, Oswaldo Cruz Foundation (FIOCRUZ), Aggeu Magalhães Research Center, Av. Moraes Rego, s/n, Campus UFPE, Cidade Universitária, Recife, PE 50670-420 Brazil; 3 Department of Biochemistry and Molecular Biology, Federal University of Viçosa, Av. Peter Henry Rolfs, s/n, Viçosa, MG 36570-000 Brazil

**Keywords:** Gene therapy, RNA interference, Adenoviral vector, *Morbillivirus*

## Abstract

**Background:**

*Canine morbilivirus (canine distemper virus*, CDV) is a highly contagious pathogen associated with high morbidity and mortality in susceptible carnivores. Although there are CDV vaccines available, the disease poses a huge threat to dogs and wildlife hosts due to vaccine failures and lack of effective treatment. Thus, the development of therapeutics is an urgent need to achieve rapid outbreak control and reduce mortality in target species. Gene silencing by RNA interference has emerged as a promising therapeutic approach against different human and animal viruses. In this study, plasmid-based short hairpin RNAs (shRNAs) against three different regions in either CDV nucleoprotein (N), or large polymerase (L) genes and recombinant adenovirus-expressing N-specific multi-shRNAs were generated. Viral cytopathic effect, virus titration, plaque-forming unit reduction, and real-time quantitative RT-PCR analysis were used to check the efficiency of constructs against CDV.

**Results:**

In CDV-infected VerodogSLAM cells, shRNA-expressing plasmids targeting the N gene markedly inhibited the CDV replication in a dose-dependent manner, with viral genomes and titers being decreased by over 99%. Transfection of plasmid-based shRNAs against the L gene displayed weaker inhibition of viral RNA level and virus yield as compared to CDV N shRNAs. A combination of shRNAs targeting three sites in the N gene considerably reduced CDV RNA and viral titers, but their effect was not synergistic. Recombinant adenovirus-expressing multiple shRNAs against CDV N gene achieved a highly efficient knockdown of CDV N mRNAs and successful inhibition of CDV replication.

**Conclusions:**

We found that this strategy had strong silencing effects on CDV replication in vitro. Our findings indicate that the delivery of shRNAs using plasmid or adenovirus vectors potently inhibits CDV replication and provides a basis for the development of therapeutic strategies for clinical trials.

**Supplementary Information:**

The online version contains supplementary material available at 10.1186/s12917-020-02671-2.

## Background

*Canine morbilivirus (canine distemper virus*, CDV) is a highly contagious and widespread virus that produces severe multisystemic disease in a broad range of wild and domestic carnivores [1]. It is the causative agent of canine distemper (CD) is an enveloped single-stranded negative-sense RNA virus and member of the genus *Morbillivirus* genus within the family *Paramyxoviridae*, closely related to the *Measles virus* (MeV) [2]. Infection with CDV is of extreme concern not only because the high mortality rates of domestic dogs [3], but also due to the large number of deaths in several endangered species [4, 5]. Additionally, CDV causes lethal infections in nonhuman primates [6], pointing to a possible threat for humans [7].

The CDV genome comprises six genes (N, P, M, F, H, and L) and codes for at least eight distinct proteins. The viral envelope contains two surface glycoproteins: hemagglutinin (H), required for cell receptor binding, and the fusion (F) protein, responsible for entry into host cells. Surrounded by the envelope, the matrix (M) protein plays a key role in CDV particle assembly and links envelope and nucleocapsid proteins. The viral ribonucleoprotein complex comprises the RNA genome combined with nucleoprotein (N), large polymerase (L), and phosphoprotein (P) [8]. While the H gene displays the highest genetic and antigenic variation [9], both the N and L genes share high degrees of sequence identity among different CDV strains [10].

Immunosuppressive mechanisms of CDV are caused by a profound viral lymphotropism that leads to an increased host susceptibility to other disease agents [11]. At the same time, both cellular and humoral immunity are strong mediators of viral clearance. Humoral immune response plays an important role in regulating the severity of the infection disorders [12]. Neurological disease may occur simultaneously or in the absence of systemic signs [13]. In the acute stage of CNS infection, demyelinating lesions are associated with CDV replication in the white matter [14]. Two mechanisms could be responsible for demyelination pathogenesis in the early phase of canine distemper. One hypothesis comprises the massive direct damage of myelin and myelin-producing cells (primary demyelination lesions). Another possible explanation describes demyelinating activity as an indirect result of CDV infection. Infected microglia cells exhibit upregulation of MHC ll and CD44 adhesion molecules in white matter, along with downregulation of GABA receptors, which may disrupt GABA functions and contribute to the demyelination process in the acute stage of CDV neurological infection [15, 16].

Extensive immunization efforts have dramatically reduced the incidence of CDV in domestic dogs. However, CDV outbreaks in dogs and wildlife have been reported repeatedly, including dog populations with high rates of vaccine coverage [17–19]. There are high mutation rates for CDV, leading to changes in surface amino acids and resulting in antigenic divergence between vaccine and wild-type strains [20–22], which may be associated with some vaccine failures. These mutations also cause alterations of receptor binding specificity related to species barriers [6].

In spite of the threat for dogs and wild carnivore species, there is no effective treatment option against CDV. Gene therapy based on RNA interference (RNAi) is emerging as a new potent therapeutic strategy against several viruses. When introduced or expressed inside the eukaryotic cells, double-stranded RNAs (dsRNAs) are cleaved into small interfering RNAs (siRNAs) of 21–23-long nucleotides. The small RNA duplexes are either delivered exogenously or processed from short hairpin RNAs (shRNAs) [23].

Intracellular siRNAs trigger a highly conserved mechanism of post-transcriptional gene silencing (PTGS) by activating the RNA-induced silencing complex (RISC). The RNA duplex is unwound into two single-stranded RNAs (ssRNAs), and one of the two strands, known as the guide strand, is incorporated into the RISC. The guide strand then directs the multicomponent nuclease complex to recognize complementary sequences on intracellular mRNAs. The binding of activated RISC to a homologous mRNA results in cleavage or translation repression of target mRNA [24].

The RNAi has been widely used to inhibit replication of several viruses, including *Human immunodeficiency virus* [25]*; Influenza virus* [26], SARS coronavirus [27], *Hepatitis C virus* [28], *Respiratory syncytial virus* [29], and *West Nile virus* [30]. With regard to morbilliviruses, studies with RNAi have shown inhibition of the MeV [31], *Peste-des-petits-ruminants virus* (PPRV), and *Rinderpest virus* (RPV) replication [32, 33].

The ShRNAs can be delivered using a plasmid or viral-based vector driven by an RNA polymerase lll or polymerase ll promoter. Although plasmid-based shRNA delivery can display effective gene-silencing activities, the most efficient shRNA delivery systems available for clinical application are based on recombinant viruses [34]. Among the viral vectors, the replication-defective human adenovirus vectors have become one of the most preferred shRNA-delivering systems for the following reasons: (i) the ability to achieve high virus titers; (ii) the wide range of cell tropism, including non-mitotic cells; and (iii) a considerably long period of transgene expression [35]. These vectors have been satisfactorily employed against numerous viruses [36–39], including the morbilliviruses MeV [37] and PPRV [38].

Emergence of resistant viruses corresponds to an important barrier for gene silencing of RNA viruses with high mutation rates. Holz et al. [39] have shown the generation of morbillivirus (PPRV) escape mutants with mutations in conserved regions of the N gene. The PPRV escaped from RNAi treatment after 3 to 20 consecutive passages, except when three different siRNAs have been simultaneously used. To prevent viral escape mutants and to improve the silencing efficiency against CDV, we developed a recombinant adenovirus vector expressing three shRNAs driven by three U6 promoters. The target gene of the adenovirus system was determined after evaluation of silencing effects of plasmid-encoded shRNAs targeting three different regions of N- and L-specific mRNAs. Here, we present a high inhibitory efficacy of plasmid-based and multi-target adenovirus-expressed shRNAs on CDV replication. To the best of our knowledge, our approach is the first to demonstrate RNAi effects against CDV. Our experimental results suggest RNAi therapy as a tool for the inhibition of CDV.

## Results

### Design of shRNAs targeting the nucleoprotein and the large polymerase genes

Multiple alignments were performed for partial (49 N and 8 L segments) and complete (23 N and 26 L genes) sequences of CDV N and L genes, and most conserved regions were identified. Three different shRNAs were designed for each conserved target (Additional file 2). Control shRNA codes for a scrambled sequence that is not complementary to any gene in the virus or in the target host. The predicted secondary structure of the designed shRNAs expressed by the human RNA pol lll promoter U6 as well as the thermodynamic stability (∆G, Kcal/mol) of shRNA duplexes are shown in Additional file 1.

### Silencing activity of N-specific shRNAs on CDV replication

**Fig. 1:**
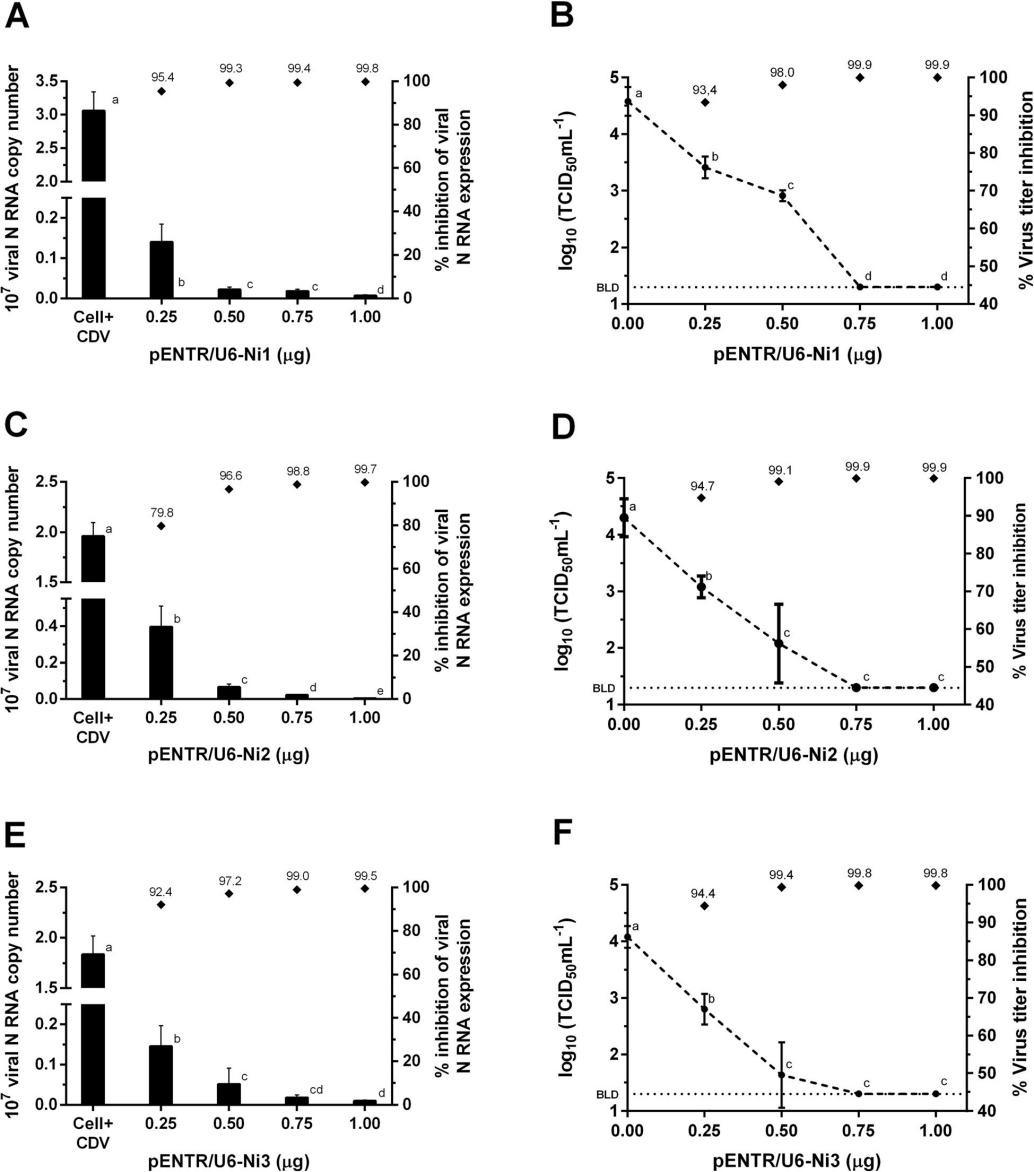
Inhibition of CDV replication by N-targeted shRNA plasmid vectors. Real-time qRT-PCR (**a**, **c**, **e**) and TCID_50_ infectivity titration (**b**, **d**, **f**) of the three N-specific plasmid-based shRNA transfections (72 h). Right vertical axis presents the percentage of virus inhibition highlighted on the markers (♦). The error bars represent standard deviations. Values given are the mean ± standard error obtained from three independent experiments. Values followed by the same lower case letters do not differ by Tukey’s test (*p* < 0.01). BLD, below limit of detection (20 TCID_50_/mL)

To demonstrate N-specific silencing effects of shRNAs under control of the U6 promoter, we constructed three shRNA-expressing plasmids targeting different conserved regions of the N gene and tested their efficacy in VerodogSLAM cells. Transfection conditions were optimized using a plasmid expressing the enhanced GFP (pEGFP) (data not shown). Negative controls for shRNA assays included non-transfected infected cells and mock-transfected cells. Results of RNA absolute quantification and TCID_50_ infectivity titration of the three N-specific plasmid-based shRNA transfections are presented in Fig. 1. The results demonstrate a remarkable silencing efficacy for all shRNA constructs directed against the CDV N gene. The target-specificity of shRNAs and the reduction in N transcript levels were determined using real-time absolute quantification. By this method, N gene expression was inhibited by up to 99.8, 99.7, and 99.5% at the highest dose used (1 μg) for Ni1, Ni2, and Ni3 shRNA transfectants, respectively (Fig. 1). This inhibition was dose-dependent. The Ni1 and Ni3 constructs showed a reduction by over 1 log (90%) in N mRNA levels, even at the lowest dose used (0.25 μg). The highest silencing efficacy was observed for Ni1 shRNA transfection, which decreased by 2.65 log of N transcript expression (Table 1). Although there was a significant reduction in N mRNA levels following transfections with Ni-shRNAs, it was important to verify if this effect would be translated into infectious virus titer inhibition. While in infected controls (no shRNA), the cell monolayer was almost entirely destroyed, we observed no CPE or detectable infectious viral particles in cells transfected with the two highest doses of Ni-shRNAs (0.75 and 1 μg). All anti-N shRNA constructs produced dose-dependent inhibition by over 99% in virus titers. Even for the dose of 0.25 μg, all N-specific transfectants showed inhibition by over 93% of viral progeny (Fig. 1). The Ni1, Ni2, and Ni3 shRNA treatments were able to reduce up to 3.28, 3, and 2.78 log10 of the virus titer, respectively.

**Table 1:** Effects of transiently pENTR/U6-expressed shRNAS on CDV replication

shRNA construct	Log_**10**_ reduction value^**a**^ and CPE score^**b**^											
	pENTR/U6-shRNA (μg)											
	0.25			0.5			0.75			1		
	qRT-PCR	TCID_**50**_	CPE	qRT-PCR	TCID_**50**_	CPE	qRT-PCR	TCID_**50**_	CPE	qRT-PCR	TCID_**50**_	CPE
**Ni1**	1.35^a^	1.17^a^	3.0	2.14^b^	1.67^b^	2.0	2.23^b^	3.28^c^	1.3	2.65^c^	3.28^c^	1.0
**Ni2**	0.71^a^	1.22^a^	3.0	1.48^b^	2.22^b^	2.3	1.92^c^	3.00^b^	1.8	2.53^d^	3.00^b^	1.0
**Ni3**	1.12^a^	1.28^a^	3.0	1.63^b^	2.44^b^	2.3	2.03^bc^	2.78^b^	1.3	2.29^c^	2.78^b^	1.0
**Li1**	ns	ns	4.0	0.29^a^	0.61^a^	3.0	0.27^ab^	0.50^a^	3.0	0.41^b^	0.78^a^	2.0
**Li2**	0.26^a^	0.50^a^	3.0	0.36^ab^	0.56^a^	3.0	0.39^b^	0.50^a^	3.0	0.31^ab^	0.56^a^	2.3
**Li3**	ns	ns	4.0	0.12^a^	ns	3.0	0.17^a^	ns	3.0	0.27^b^	0.72^a^	2.0
**SCR**	ns	ns	4.0	ns	ns	4.0	ns	ns	4.0	ns	ns	4.0
**Ni shRNAs association**^**c**^	**Ni1 (1.2 μg)**			**Ni2 (1.2 μg)**			**Ni3 (1.2 μg)**			**Ni1 + Ni2 + Ni3 (0.4 μg each)**		
	2.54^b^	3.1^a^	1.0	1.90^a^	3.1^a^	1.0	1.92^a^	3.1^a^	1.0	1.87^a^	3.1^a^	1.0

^a^The log_10_ reduction was calculated by subtracting the log_10_ means of the CDV infectivity in the presence of transfected shRNA constructs from the log_10_ means of the CDV infectivity in the absence of shRNAs

^b^CPE scores were defined as (1) CPE < 25%, (2) CPE between 25 and 50%, (3) CPE between 51 and 75% and (4) CPE > 75%. CPE values correspond to the CPE score means for replicates

^c^Association of pENTR/U6-shRNAs against CDV nucleoprotein

Values followed by the same lowercase letters in horizontal lines do not differ within each method; *p* < 0.01 (Tukey’s test)

*Ni* plasmid shRNA for CDV nucleoprotein mRNA, *Li* plasmid shRNA for CDV large protein mRNA, *SCR* plasmid scramble shRNA, *ns* (not statistically significant): no significant difference between shRNA-treated and untreated CDV infected cells

Silencing efficiency was also demonstrated by the marked decrease in CPE. Table 1 shows the dose-dependent reduction of CPE scores for N-specific shRNA transfections. As expected, control scrambled shRNA delivered using pENTR/U6-SCR displayed no meaningful suppressive activity against CDV N gene expression or viral replication (Additional file 5). Taken together, quantitative analyses of CDV N mRNA levels and infectious virus titration of Ni-construct transfections showed strong silencing effects on CDV replication.

### Silencing activity of L-specific shRNAs on CDV replication

**Fig. 2:**
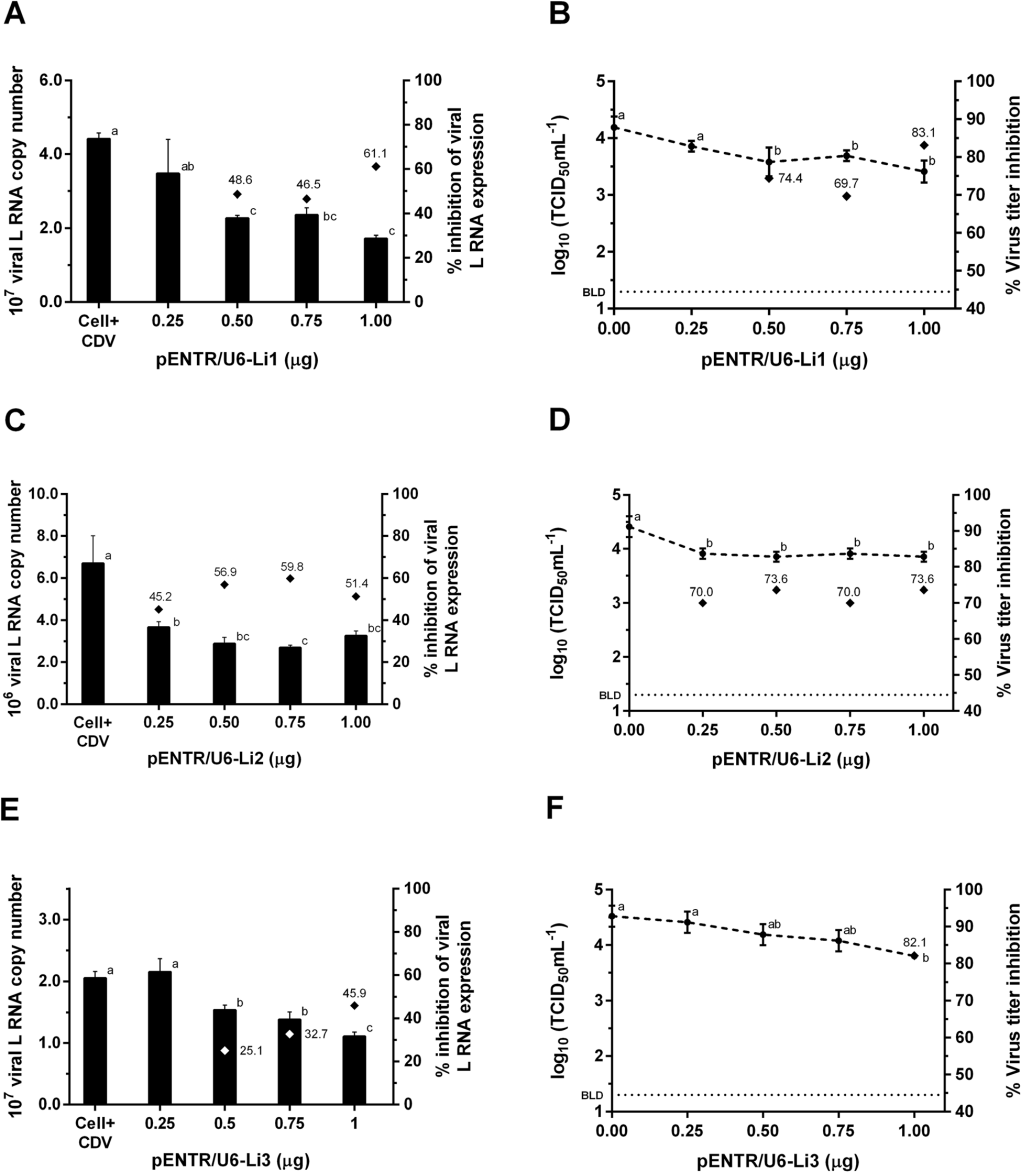
Inhibition of CDV replication by L-targeted shRNA plasmid vectors. Real-time qRT-PCR (**a**, **c**, **e**) and TCID_50_ infectivity titration (**b**, **d**, **f**) of the three L-specific plasmid-based shRNA transfections (72 h). Right vertical axis presents the percentage of virus inhibition highlighted on the markers (♦). The error bars represent standard deviations. Values given are the mean ± standard error obtained from three independent experiments. Values followed by the same lower case letters do not differ by Tukey’s test (*p* < 0.01). BLD, below limit of detection (20 TCID_50_/mL)

To investigate whether the CDV L gene would be a good target for the RNAi strategy, we generated plasmid-expressed shRNAs against three different regions of the L mRNA. Inhibition assays were conducted as described before for the N gene. Silencing activities of the three shRNA-expressing plasmids against the L gene are shown in Fig. 2 as RNA copy numbers and TCID_50_/mL. The Li1, Li2, and Li3 plasmids suppressed L mRNA expression in a dose-dependent manner, but none of these shRNAs achieved 1 log_10_ of mRNA level reduction (Table 1). The levels of L mRNA were partially reduced up to 61%. L-directed shRNA treatments led to maximum reductions of the infectivity titer of 83% by Li1, 73.6% by Li2, and 82% by Li3 (Fig. 2a, c, e). Similar to the results obtained for the absolute quantification of target transcripts described above, Li shRNAs titration did not achieve 1-log_10_ reduction (Fig. 2b, d, f; Table 1). As expected, the scrambled shRNA plasmid vector did not reduce L mRNA or infectious titers (Fig. 2b, c). Virus titration results and CPE scores (Table 1) of pENTR/U6-Li shRNAs also confirmed its weaker inhibitory effects as compared to Ni-shRNA treatments.

### Co-transfection using multiple shRNA-plasmid vectors against CDV N gene

**Fig. 3:**
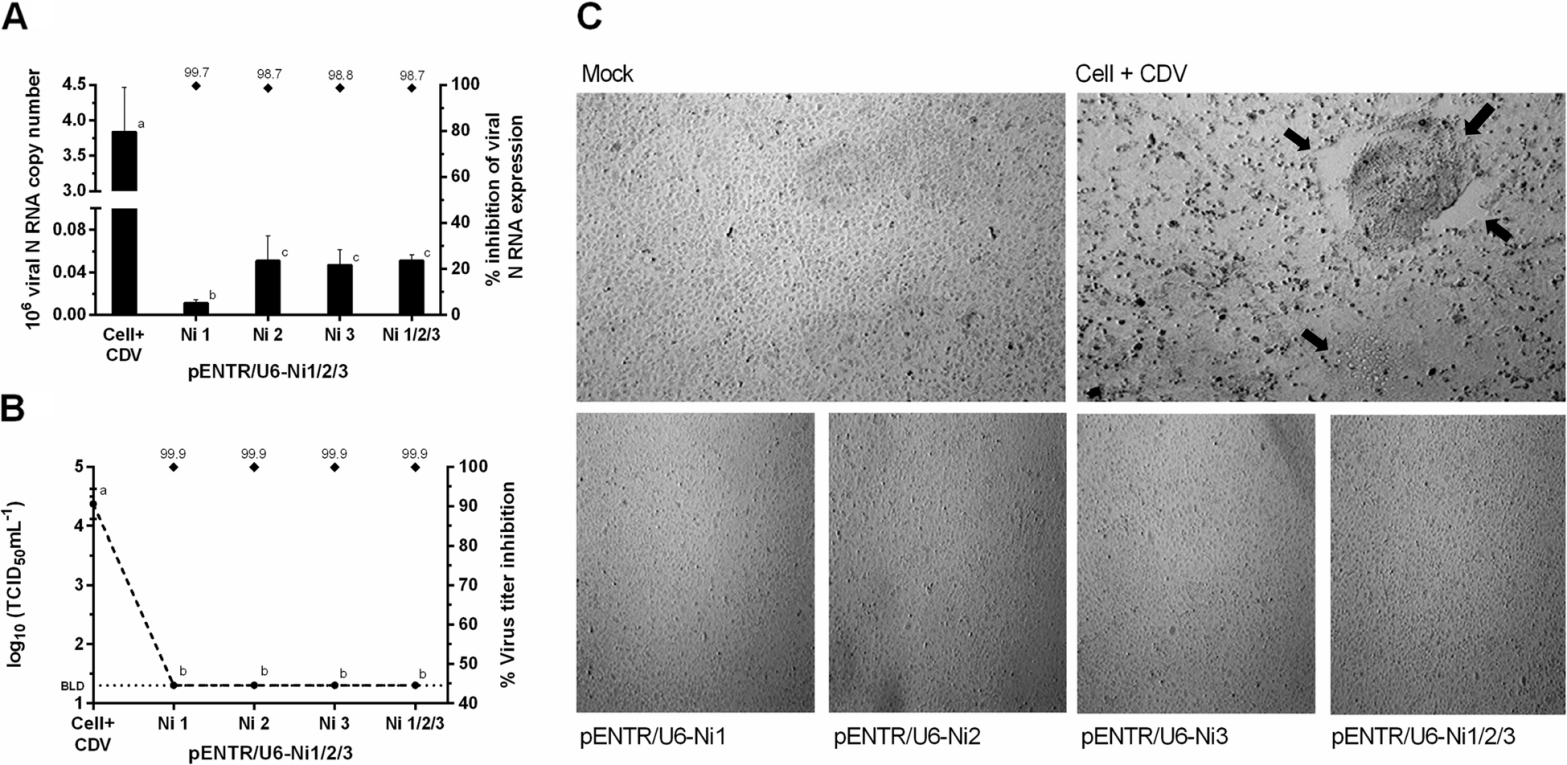
Co-transfection of shRNA-expressing plasmids targeting nucleoprotein gene. VerodogSLAM cells were first infected with CDV MOI 0.01 and then transfected with 1.2 μg of single or combinated pENTR/U6-Nis (0.4 μg each). Combination of the three N-specific shRNA plasmid vectors exhibited markedly reduced N transcript levels (**a**), infectious virus titers (**b**) and CPE (**c**). Average and standard deviations represent three independent transfections. Right vertical axis presents the percentage of virus inhibition highlighted on the markers (♦). More evident cytopathic effects are indicated by arrows. Values followed by the same lower case letters do not differ by Tukey’s test (*p* < 0.01). BLD, below limit of detection (20 TCID_50_/mL). All figure panels (100X total magnification): representative data from at least three independent experiments using biological replicates

We analyzed the results obtained for shRNA silencing efficacy against the two target genes. The N-specific shRNA treatments showed markedly stronger antiviral effects than that displayed for shRNA plasmids against the L gene. We sought to enhance the antiviral effects by simultaneously expressing three shRNAs against the N gene. Co-transfection of N-directed shRNAs was performed with 0.4 μg of each pENTR/U6-Ni construct into VerodogSLAM cells infected with CDV. Silencing effects of multi-transfected shRNAs against the N gene were compared with 1.2 μg of each N-targeted shRNA individually transfected into infected cells. As shown in Fig. 3a, Ni-shRNA combination was highly efficacious and exhibited roughly 99% reduction of N mRNA levels. However, the triple Ni-shRNA treatment showed no improvement in the antiviral effect against CDV compared to single transfections of N-specific shRNAs. Moreover, individually expressed Ni1-shRNA resulted in a higher suppression as compared to the combinatorial strategy. The infectious titer of combinatorial shRNAs on CDV was decreased by over 3 log_10_ (> 99.9%), and there was no significant difference when compared with the single transfected Ni-shRNAs (Fig. 3b; Table 1). Indeed, no CDV infective particles were detected for either combined or single shRNA transfections, as exhibited in Fig. 3c and demonstrated for CPE score analysis (Table 1).

### Ad5-shRNA construction and its impact on cell viability

Based on the results obtained from plasmid-based shRNA against CDV N and L genes, we developed a recombinant human adenovirus serotype 5 (Ad5) that simultaneously expresses three different shRNAs targeting the N gene under the control of independent U6 promoters. Recombinant adenovirus DNA was confirmed by PCR. Adenoviral vectors were amplified in 293 cells (two passages), and virus titrations were carried out by TCID50. The Ad5Ni(1–3) and Ad5SCR P2 viral titers were 2 × 10^8^ TCID50/mL and 1.8 × 10^7^ TCID50/mL, respectively.

We analyzed the viability of the VerodogSLAM cells transduced by Ad5Ni(1–3) at different MOI values using the MTT assay. In this study, the cytotoxic concentration of 20% of the cell culture (CC_20_) was defined as the limit point for treatment with recombinant adenovirus. Control Ad5SCR cytotoxicity was also evaluated by the MTT assay. The MOI values of Ad5Ni(1–3) and Ad5SCR corresponding to CC_20_ were 31.6 ± 2.9 and 69.9 ± 1.4, respectively. These results demonstrate that adenovirus expressing multiple shRNAs had increased cell toxicity compared with the control Ad5 vector encoding scrambled shRNA. Toxic effects in cultured cells were dose-dependent and correlated with the number of transcribed shRNAs (Additional file 6).

### Antiviral effects of N-targeted multiple shRNA-expressing adenovirus vector

**Fig. 4:**
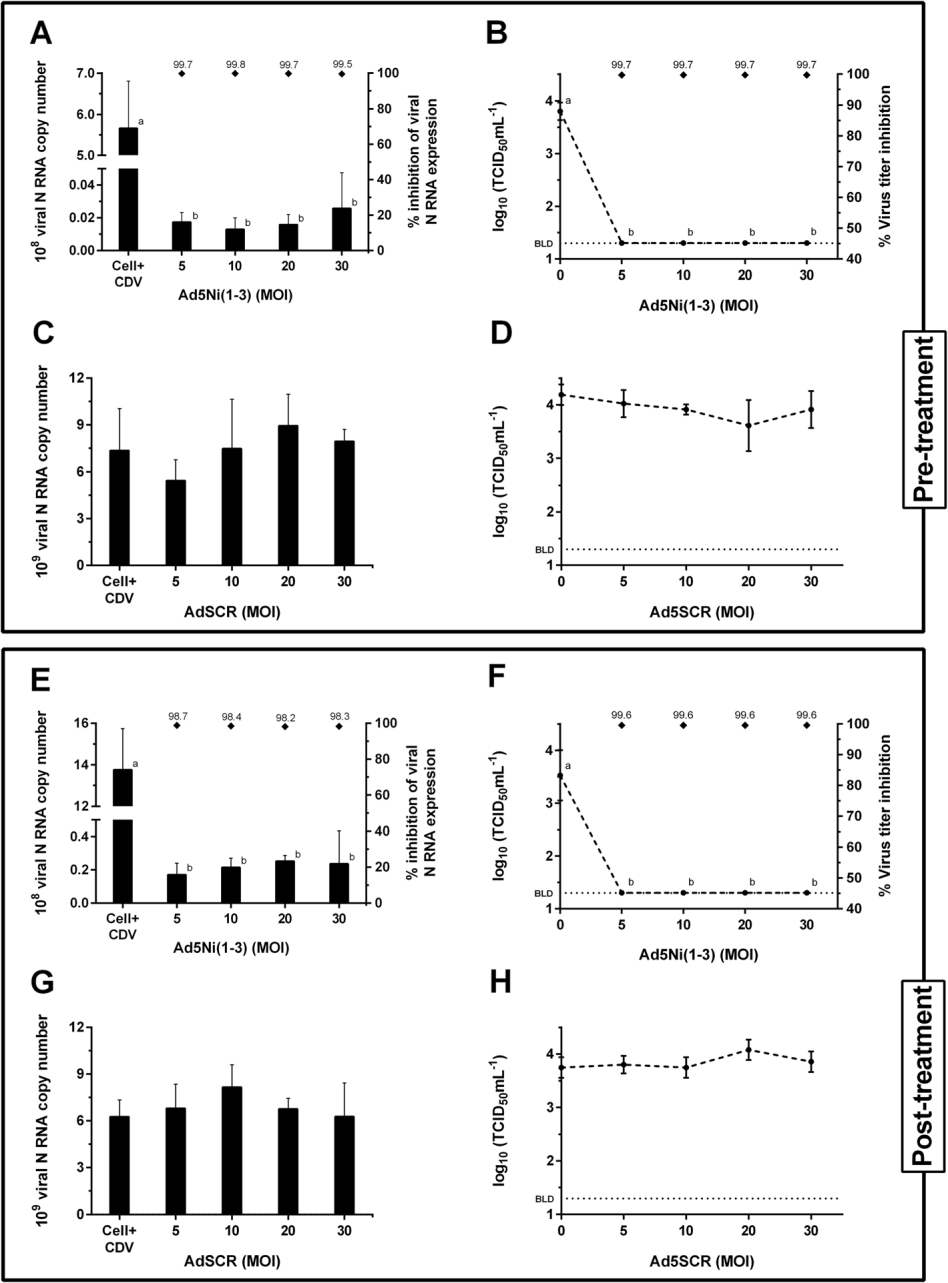
Antiviral effects by pre-treating or post-treating CDV-infected cells with multi-shRNA Ad5 construct at various doses. Cell+SUP were collected 72 hpi for detection of N mRNA levels (**a**, **c**) and viral titers (**b**, **d**). Ad5SCR was used as negative control. Values are the mean ± standard error obtained from three independent experiments. Right vertical axis presents the percentage of virus inhibition highlighted on markers (♦). Values followed by the same lower case letters do not differ by Tukey’s test (*p* < 0.01). BLD, below limit of detection (20 TCID_50_/mL)

**Fig. 5:**
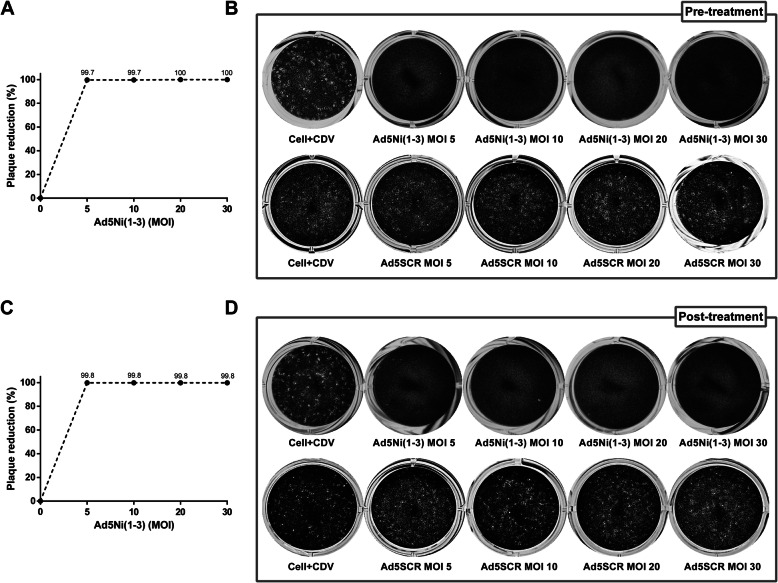
Transduction of VerodogSLAM cells with Ad5Ni(1–3) vector blocks CDV plaque formation. Samples of Ad5 construct pre- and post-treatments were added to cells and after 2-h incubation, the inoculums were removed, cells were overlaid with CMC complete medium and incubated for 72 h. Plaque reduction values of Ad5 pre-treating (**a**, **b**) and post-treating (**c**, **d**) assays represent the mean ± standard error from three independent experiments compared with untreated infected cells. Ad5SCR was used as negative control. (**b**, **d**) CDV-plaque crystal violet-staining method

Since the Ad5Ni(1–3) dose limit for minimal cellular toxicity was assessed, dose-response analyses were performed with increasing MOI levels (5, 10, 20, and 30) of the multi-shRNA adenoviral vector in subsequent experiments. For this, VerodogSLAM cells were treated with Ad5Ni(1–3) before or after CDV infection, and the viral infectivity and CDV RNA levels were measured by TCID_50_ assay and qRT-PCR, respectively. In the pre-treatment experiment, Ad5Ni(1–3) was transduced to the cells 12 h prior to CDV infection (MOI 0.01) and displayed remarkable levels of inhibition. There was a significant reduction in CDV RNA copy number, as much as 99.7%, and no virus particles were detected by the TCID_50_ assay (Fig. 4a and b). The differences in RNA level and virus titer among all four Ad5Ni(1–3) MOI were not statistically significant. In the post-treatment experiment, cells were first infected with 0.01 MOI of CDV and then inoculated with four different MOI levels of Ad5Ni(1–3). Levels of virus RNA were reduced by more than 98%, and there was no infectious titer measurable for all Ad5Ni(1–3) MOI treatments (Fig. 4c, d). Although Ad5Ni(1–3) pre-treatment presented a slightly higher reduction in CDV RNA, both pre- and post-treatments induced a highly efficacy inhibition of viral replication. Plaque assays showed that pre-treatment with Ad5Ni(1–3) completely abolished CDV plaque formation (Fig. 5a, b), whereas Ad5Ni(1–3) post-treatment resulted in 99.8% inhibition of CDV-infectious plaque (Fig. 5c, d). To eliminate the possibility that pre- and post-treatment silencing effects were due to a non-specific outcome of pre-treating or challenging CDV-infected cells with recombinant adenovirus, we inoculated Ad5SCR at the same MOI and conditions outlined above for both experiments. As expected, there was no significant inhibition for CDV RNA levels, virus titers, and CDV-plaque number (Fig. 4a, b, c, d; Fig. 5b, d).

**Fig. 6:**
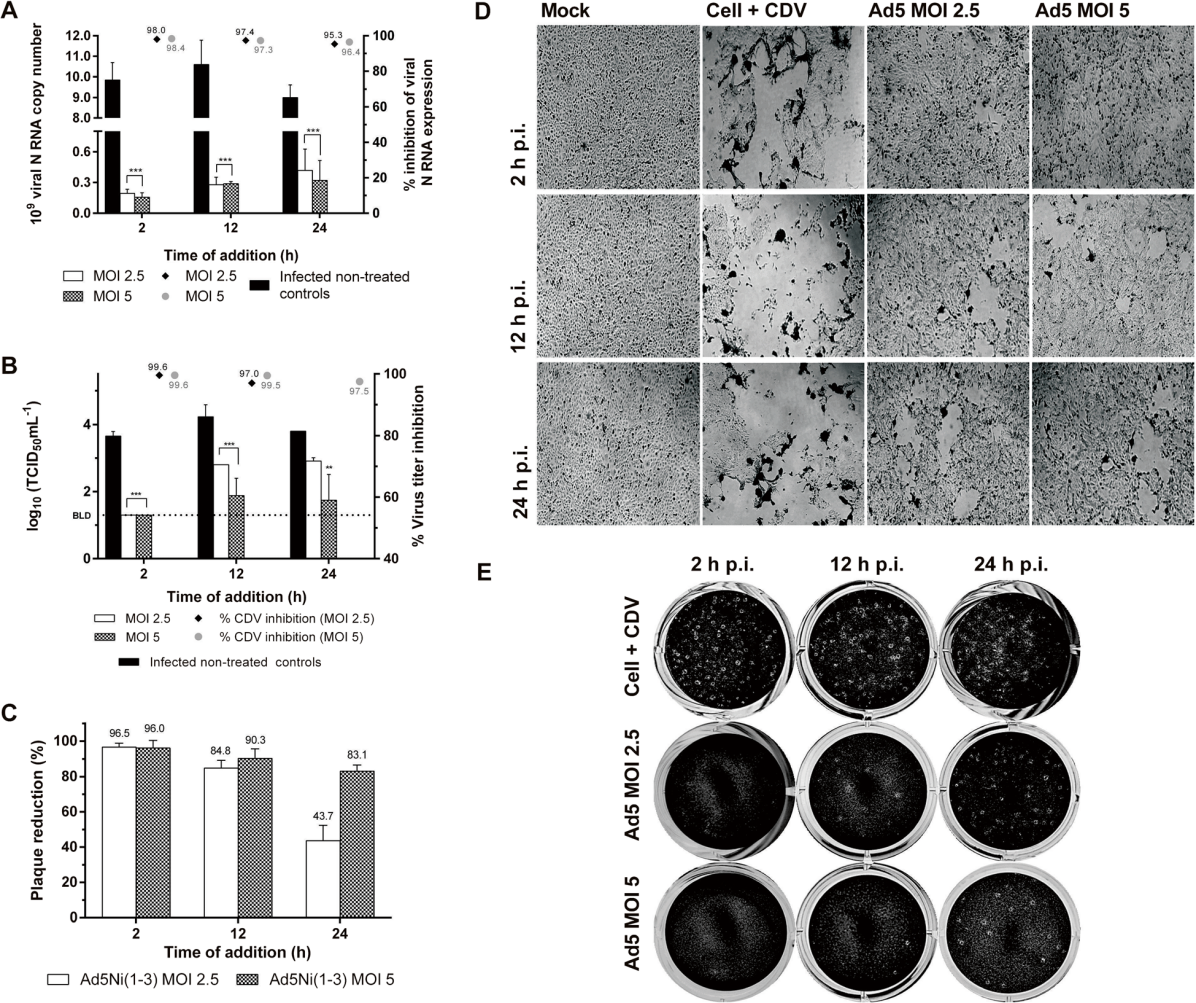
Ad5Ni(1–3) post-treatment at various time intervals. Multi-shRNA Ad5 construct was added at MOI of 2.5 and 5 to infected cells at 2, 12 and 24 hpi. Ad5 silencing efficiency was measured by viral RNA levels (**a**), infectious titers (**b**), and CDV plaque count (**c**) at time-specific treatments (** *p* < 0.01; *** *p* < 0.001). CDV infectivity inhibition was shown through reduction of CPE (D, 100X total magnification) and infectious plaque number (**e**) compared to controls

After successfully demonstrating the antiviral activity by post-treating with Ad5Ni(1–3), we investigated the silencing effects of inoculating Ad5Ni(1–3) at various time intervals following CDV infection (2, 12, and 24 h) (Fig. 6). Since there was statistically significant difference for CDV inhibition at different Ad5-vector MOI values of pre- and post-treatment, we decided to employ the MOI values of 5 and 2.5 in the time-of-addition assay. Treatment at 2 h post-infection showed the greatest silencing efficiency, and there was no significant difference between the Ad5-vector MOI values used. The viral RNA transcripts decreased by about 98%, and that result was followed by over 99.6% CDV infectivity inhibition and roughly 96% reduction of viral plaque count (Fig. 6). At 12 h post-infection (pi), RNA copy number and virus replication levels started to increase, but the values remained lower compared to the control. We observed a 97.3% reduction of the CDV RNA level for both Ad5Ni(1–3) MOI levels. Infections titer inhibition was 97 and 99.5% with 2.5 and 5 MOI of Ad5-vector, respectively. The plaque reduction assay resulted in about 90% reduction of the CDV-infectious plaque number, and no significant difference was reported between MOI values. Although presenting a lower treatment efficacy, transduction with Ad5Ni(1–3) at 24 hpi maintained its high silencing activity at the MOI of 5. Despite the RNA level reduction of around 95%, the MOI of 2.5 resulted in only 43.7% reduction of viral plaque count and produced no significant inhibition of the infectious virus titer. Our findings suggest that the earlier the Ad5Ni(1–3) treatment of CDV-infected cells, the higher the inhibition of virus replication.

## Discussion

Here, we developed for the first time RNAi-based strategies aimed at suppressing CDV replication. We showed that N-targeted shRNA plasmids induced a highly efficacious inhibition of CDV replication. The reduction of the target mRNA level indicated that gene silencing occurred through RNAi-induced mRNA cleavage instead of translational repression. The silencing efficacy of the three generated plasmid-expressed shRNAs targeting the L gene was comparatively lower than that for the N gene. While Ni-shRNAs plasmids achieved titer reductions of up to 3.3 log_10_, Li-shRNAs did not reduce CDV titers to more than 0.8 log_10_ (Table 1). The CDV RNA polymerase, which is encoded by the L gene, is the largest but the least abundant of viral proteins. It is possible that even at partially inhibited level, its presence at low levels would be able to maintain virus replication. The relative inefficacy of L-targeted shRNA plasmid vectors could also be attributed to virus mutations in the corresponding gene [40], the presence of double-stranded (ds) RNA-binding proteins [41], or to intramolecular secondary and tertiary structures of the targeted mRNA, since RNAi recognition and binding to the complementary region depend on the target structural features for shRNA functionality [42]. A study published by Almeida et al. [32] suggested that *Morbillivirus* RNA hairpin structures and target sequences placed among two close branches may restrict its accessibility and propitiate unsuccessful siRNA activity.

Several reports have shown siRNA effects on *Morbillivirus* gene expression, and most of these studies were based on silencing approaches targeting the N gene. For example, Keita et al. [43] designed siRNAs against conserved N regions among morbilliviruses and reported a consistent inhibition of PPRV, RPV, and MeV replication. The large polymerase corresponds to another well-conserved morbillivirus gene and was selected for some RNAi studies. In this way, synthetic and plasmid-mediated siRNAs targeting the L gene efficiently suppressed MeV replication [37]. Furthermore, Reuter et al. [44] demonstrated that multiple siRNA sequences directed to six target genes of MeV resulted in a higher silencing efficacy of N and L expression compared to the other genes. Shi et al. [45] also found a strong inhibitory efficacy for siRNA-expressing plasmid therapy against N and L genes of MeV. Overall, most of *Morbillivirus* silencing studies indicated N and L genes as good targets for RNAi approaches. In agreement with these studies, we showed that N-silencing expression plasmids inhibited nearly 100% of CDV replication. However, we did not observe the same levels of silencing activity for L-targeted shRNAs. In accordance with this finding, Leber et al. [46] incorporated microRNA target sites (miRTS) into the MeV genome, showing that L gene silencing is less prejudicial to viral replication when compared with N gene silencing.

There has been an increasing concern about the risk of viral escape mutants restricting RNAi activity, and RNA viruses generally display high mutation rates that could lead to the emergence of RNAi-resistant mutants. Although the N gene corresponds to the most conserved region, Holz et al. [39] demonstrated PPRV adaptive responses to siRNA therapy directed to well-conserved regions in this gene. The RNAi-escaping mutants acquired resistance to single or multiple siRNAs, except for the simultaneous use of three siRNAs. In this context, to further overcome the emergence of escape mutants and to enhance the silencing efficiency, we co-transfected CDV-infected cells using the three N-specific plasmid-based shRNAs, which previously showed the highest inhibitory efficacy on single transfection assays. Combinatorial RNAi strategies have been effectively employed against *Human immunodeficiency virus* (HIV) [47] and *Hepatitis C virus* (HCV) [48]. In our study, Ni-shRNA combination was highly efficient, but we did not observe synergistic effects based on N-transcript quantification. Although Ni1-shRNA transfection presented a slightly better knockdown efficiency, infectious titer was undetectable for both single and combined shRNA treatments. Since there was a decline in titers to below the detectable level, synergistic effects on CDV replication could not be confirmed. On the other hand, a possible explanation for no synergistic combination is that the mixed-shRNA transfection resulted in N gene inhibition proportional to Ni2- and Ni3-shRNA, which exhibited a slightly lower efficiency than Ni1-shRNA for single transfections. Such a transient co-transfection approach might have achieved high expression rates of shRNAs and saturated the cellular RNAi silencing pathway. Overexpression of shRNA may lead to suppression of RNAi nucleo-cytoplasmic transport and processing [49]. In this way, a high dose of vector-encoded shRNAs may imply RNAi saturation effects instead of synergistic activity. Maybe the combination strategy could have resulted in an improved efficacy if we had considered lower doses of N-targeted shRNA-expressing plasmids.

Based on the successful blockage of CDV replication using plasmid-delivered shRNA against the N gene, we engineered a replication-defective human adenovirus type 5 vector to express the combined three shRNAs targeting the N gene driven by U6 promoters. This multi-shRNA N-targeted expression cassette was designed to counteract the possible emergence of escape mutants and to improve the efficacy of gene silencing effects. Delivery of Ad5Ni(1–3) had strong antiviral effects against CDV both in pre- and post-treatment. The Ad5-vector post-treatment at various time intervals demonstrated a dose- and time-dependent silencing efficacy, which remained relatively high even for Ad5 transduction at lower MOI values and 24 h post-infection.

Kim et al. [50] demonstrated that the silencing efficiency of the Ad-vector expressing three shRNAs driven by three separated U6 promoters was higher compared with the Ad-vector expression of three shRNAs under the control of two U6 promoters and one CMV promoter. However, the improved multi-shRNA Ad-vector efficiency was associated with increased cell toxicity. Likewise, our results show that cell toxic effects were correlated with the number of Ad5-transcribed shRNAs and, consequently, dependent on shRNA abundance. Other studies have also reported a direct correlation between shRNA expression and toxic effects [49]. A possible reason for the increased cytotoxicity would be the shRNA overexpression driven by U6 RNA polymerase lll promoters. Standard pol lll promoters can synthesize high levels of shRNA, which could result in saturation of the endogenous microRNA-processing machinery, further resulting in cytotoxicity and tissue damage [49]. High-level shRNA expression may induce the interferon (IFN) pathway and result in dose- and cell type-dependent nonspecific toxicity [51]. Furthermore, shRNA-expressing adenovirus vectors have been shown to trigger IFN responses, and IFN induction is dose-dependent as well as shRNA sequence-dependent [52]. Accordingly, the Ad5-vector dose has been optimized to yield high transduction and shRNA expression rates with low cytotoxicity levels. Besides lowering the vector dose, an alternative approach would be switching U6 promoters to weaker variants, such as H1 or 7SK, to better prevent off-target effects [53].

## Conclusion

We conclude that plasmid-mediated shRNAs targeting CDV N and L genes could specifically inhibit CDV production in VerodogSLAM cells, and silencing effects of N-targeted shRNAs were markedly higher compared to those exhibited for shRNAs directed to the L gene. We also demonstrated that a recombinant adenovirus expressing multiple shRNAs against the CDV N gene achieved a highly efficient knockdown of CDV N mRNAs and successful inhibition of CDV replication. In summary, plasmid-based and adenovirus-encoded shRNAs therapeutic platforms produced strong antiviral activity against CDV in in vitro assays; thus, multi-target adenovirus-expressing shRNAs should be considered in animal model analysis.

## Methods

### Cells and virus

The VerodogSLAM cells (kindly provided by Dr. V. von Messling, Veterinary Medicine Division, Paul-Ehrlich-Institute, Germany) and human embryonic kidney cells (HEK 293) (ATCC CRL-1573) were cultivated in Dulbecco’s modified Eagle’s medium (DMEM High Glucose, Sigma) containing 10% fetal bovine serum (FBS, Gibco), 1% penicillin/streptomycin, and 1 mM L-glutamine (Gibco) at 37 °C in a humidified incubator with 5% CO_2_. Zeocin (Invitrogen) was added at 1 mg/mL to maintain selection for SLAM (Signaling lymphocytic activation molecule) expression in the VerodogSLAM cell line. The HEK 293 cells were used to generate, grow, and titer the recombinant adenovirus.

A CDV wild-type strain (CDV/LDM-BTU-2, GenBank accession number KJ865300.1), isolated from canine clinical specimens, was used in this study. The CDV/LDM-BTU-2 strain was propagated on the VerodogSLAM cells, and virus stocks were prepared by collecting the infected cells plus supernatant (sup) when CPE (cytopathic effect) was ~ 80%. The virus stock was stored in aliquots at − 80 °C, and virus titers were determined by the method of Reed and Muench (1938) and expressed as log_10_ TCID_50_/mL.

### Design and construction of shRNA expression plasmids

The CDV N and L genes were defined as virus targets for designing shRNAs because of their high degree of conservation among CDV isolates. We aligned all partial and complete sequences of CDV-selected genes available in GenBank, using the MEGA software v. 6 [54]. We chose three different 20-nt successive stretches with a high degree of similarity for each gene. Top and bottom oligos specific for each gene segment were designed using the web-based BLOCK-iT RNAi Designer (Invitrogen, USA) available at https://rnaidesigner.thermofisher.com/rnaiexpress/ and custom-synthesized by Life Technologies (USA). The chosen shRNA sequences against CDV L and N genes are summarized in the Additional files 1 and 2. Each top strand was designed to include a gene homologous region, a loop, an antisense sequence, and 5′ overhangs for cloning into the pENTR/U6 RNAi entry vector (Invitrogen). A blast search against the canine (*Canis lupus familiaris*) genome confirmed no homology between the oligo sequences and any other genes. A scrambled sequence (SCR) with no homology to any known CDV gene sequence was also generated as negative control for designed shRNAs (Additional file 1). The predicted folding of the selected shRNAs was done using the mfold web server v. 2.3 [55]. The thermodynamic stability (∆G, Kcal/mol) of the shRNA duplexes (Additional file 2), which determines the asymmetrical RISC assembly, was calculated as previously described [56].

Sense and antisense oligos were mixed, heated at 95 °C for 5 min, and allowed to anneal by slowly decreasing the temperature. Double-stranded oligonucleotide was cloned into the pENTR/U6 vector downstream of the human U6 shRNA promoter (Fig. S1B) following an established protocol (Invitrogen). Recombinant plasmids were verified by colony PCR using M13 primers and *Ndel* and *Xbal* double restriction digestion (Thermo Scientific). All constructs were sequenced to ensure the correct design. All seven resulting pENTR/U6-shRNA constructs were used for transient shRNA expression in VerodogSLAM cells and evaluation of antiviral activity.

### Transfection of shRNA constructs

The VerodogSLAM cells were seeded 1 day prior to transfection in 24-well tissue culture plates at a density of 2 × 10^5^ cells/well in DMEM supplemented with 10% FBS, so that the cell monolayer was fully confluent by the time of the transfection. After 24 h, cells were inoculated with CDV/LDM-BTU-2 at a multiplicity of infection (MOI) of 0.01. Following an incubation period of 2 h at 37 °C, the inoculum was removed and the cells were washed twice with 500 μL of DMEM. Four different amounts of plasmids (0.25, 0.5, 0.75, and 1 μg) were each complexed with 2 μl Lipofectamine 2000 (Invitrogen) per well in 100 *μ*L of DMEM serum-free medium. Plasmid DNA and Lipofectamine 2000 were mixed and incubated for 20 min at room temperature according to the manufacturer’s instructions. The infected cells were washed, and the medium was replaced with 200 *μ*L of fresh DMEM serum-free medium, followed by the addition of 100 *μ*L of plasmid-lipofectamine complexes. Six hours later, the transfection medium was replaced by 1 mL of DMEM containing antibiotics and 2% FBS. All assays were performed in triplicate. At 72 h post-infection, RNAi silencing efficacy was evaluated by observing and scoring the cytopathic effects (CPE). The CPE scoring scale was defined as follows: 1 – CPE < 25%, 2 - CPE between 25 and 50%, 3 - CPE between 51 and 75%, and 4 – CPE > 75%, as exemplified in Additional file 3. At the same time point, the plates were frozen/thawed 1X, and cell lysates plus supernatant were harvested and stored at − 80 °C until use for virus titration and real-time quantitative RT-PCR analysis. The virus titer was determined according to the method of Reed and Muench, [57] and expressed as log_10_ TCID_50_/mL. Target-specific shRNAs constructs with the highest silencing effects were co-transfected to determine whether gene suppressive activity would be enhanced.

### Construction of multiple shRNA-encoding adenoviral vector

Multiple shRNA-expression cassettes were designed with three N-specific shRNAs under control of the U6 promoter. The target gene was determined according to the suppressive activity of plasmid-based shRNAs previously mentioned. The shRNA sequences (Ni1, Ni2, and Ni3) selected for construction of the recombinant adenovirus are summarized in Additional file 1. The multi-shRNA fragment was synthesized (GenScript Corporation, Piscataway, NJ USA) with restriction enzyme sites Clal and Xbal as sticky ends and inserted into a shuttle plasmid (pUC57), using standard cloning procedures. Likewise, a scrambled shRNA control cassette containing sh-SCR (Additional file 1) was synthesized and transferred into a pUC57 vector.

**Fig. 7:**
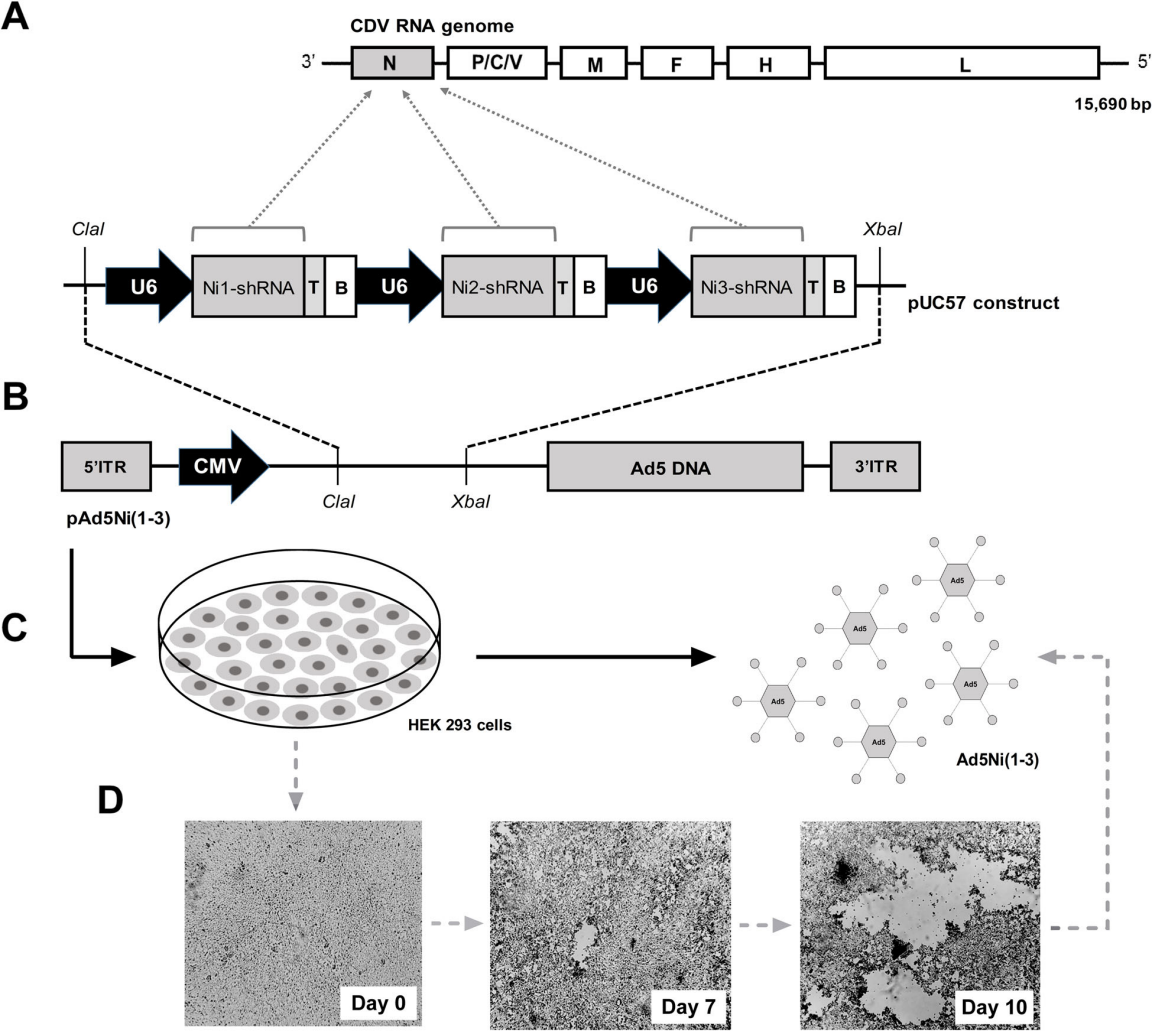
Schematic representation of multiple shRNA-expressing adenovirus construct. (**a**) The multiple shRNA-expressing cassette are transcribed under the control of U6 promoters. The cassette also includes polyT transcription termination signals (T) and spacer blocks (**b**) of 100 nt between shRNA-coding regions to prevent potential cross-interference. (**b**) Multi-shRNA expression cassette was cloned into the E1 region of an E1/E3-deleted Ad5 vector plasmid by restriction enzyme sites (*Clal-Xbal*). A recombinant adenovirus with scrambled shRNA sequence (Ad5SCR) was generated and used as a negative control. (**c**, **d**) Ad5-vector particles were produced by transfecting *Pacl*-linearized pAd5Ni(1–3) clone. HEK293 cells were transfected with pAd5Ni(1–3) and after 7–10 days, isolated plaques of recombinant Ad5 were picked to obtain the monoclonal virus, and the Ad5Ni(1–3) virus was further identified by PCR and sequencing. (**d**) Ad5Ni(1–3) transduction and cytopathic effects observed at days 7 and 10 (40X total magnification)

To generate the recombinant replication-defective *Human adenovirus type 5* (Ad5) vector, each shuttle pUC57 construct was digested with *Clal* and *Xbal* (New England Biolabs), and the fragment of interest was ligated into the corresponding sites of the pAd5-Blue vector. The DNA ligation mix was transformed into electrocompetent TOP10 cells, and the obtained colonies were screened by PCR with the primers pAd5 forward 5′-AAGTGTGGCGGAACACATGTAAGC-3′ and pAd5 reverse 5′-AAGCAAGTAAAACCTCTACAAATGTGGTATGG-3′, which flank the transgene. Plasmid DNA minipreps were digested with *Hindlll* (New England Biolabs) and compared with the vector control pAd5-Blue to verify the integrity of the Ad5 constructs and to identify which minipreps contained the desired transgene. Plasmids were also digested with *BsrGl* (New England Biolabs) and sequenced in an ABI PRISM® 3100 Genetic Analyzer to confirm their correctness. The Ad5 recombinant plasmids were linearized with *Pacl* (New England Biolabs), purified by ethanol precipitation, and transfected into low-passage HEK 293 cells that had been plated the previous day. The HEK 293 cells cultured in 6-well plates at 30% confluence were transfected with the linearized adenoviral plasmids using PolyFect Transfection Reagent (Qiagen) and visually monitored for CPE (cell rounding and detachment). Recombinant viruses were harvested 7–10 days after transfection upon the appearance of CPE, amplified in HEK 293 cells, and confirmed by PCR (pAd5 forward and reverse) and restriction analyses (*Hindlll* digestion). The Ad5 vector constructs expressing N-specific multi-shRNAs and scrambled shRNA were defined as Ad5Ni(1–3) and Ad5SCR, respectively. A schematic representation of the recombinant viruses is represented in Fig. 7. Virus titration was performed with serial 10-fold dilutions of recombinant adenoviruses and expressed in log_10_ TCID_50_/mL as described by Reed and Muench [57].

### Assessment of cell viability after Ad5 vector infection

Cell toxicity of recombinant Ad5Ni(1–3) and Ad5SCR was determined using a colorimetric method which is based on the mitochondrial reduction of 3-(4,5-dimethylthiazol-2-yl)-2,5-diphenyltetrazolium bromide (MTT) (Sigma, St. Louis, MI). Briefly, VerodogSLAM cells were plated at a density of 1 × 10^4^/well in 96-well microplates and inoculated 24 h later with DMEM-diluted Ad5 constructs at MOI values of 10, 20, 30, 40, 50, 100, 200, and 300. After a 1-h adsorption period at 37 °C, the inoculums were removed and cells were washed twice with DMEM. Subsequently, 200 uL of DMEM with 2% FBS were added and cells were incubated for 72 h at 37 °C. At 72 h post-transduction, the culture medium was removed and 50 μL of MTT working solution (1 mg/mL) were added to each well; the microplate was incubated for 4 h. The MTT formazan crystals were solubilized by adding DMSO, and the optical densities were determined by an absorbance microplate reader (ELX800 Absorbance Microplate Reader, BioTek, Winooski, Vermont, USA) with a 540-nm optical filter. Cell viability was calculated by subtracting the optical density fraction of treated cells from the untreated cells. At least three independent experiments were performed, with eight replicates per treatment.

### Pre-treatment with the recombinant Ad5 construct

The VerodogSLAM cells were seeded 1 day prior to the experiment in 24-well tissue culture plates at a density of 10^5^ cells/well in DMEM supplemented with 10% FBS. Cells were transduced with Ad5Ni(1–3) at MOI values of 5, 10, 20, and 30, and after a 1-h adsorption period at 37 °C, the inoculums were removed, cells were washed twice with DMEM, and the medium was replaced by culture medium containing 2% FBS. After an incubation time of 12 h, cells were infected with CDV/LDM-BTU-2 at the MOI of 0.01 and incubated for 2 h at 37 °C and 5% CO_2_. The viral inoculum was removed and cells were washed twice with DMEM and replaced by culture medium with 2% FBS. Mock and CDV infection controls (no Ad5 construct pre-infection) were set up in parallel. Recombinant adenovirus with scrambled shRNA sequence was also evaluated under the same conditions. At 72 h post-inoculation, 24-well plates were frozen/thawed 1X, and cell lysates plus supernatant were harvested and stored at − 80 °C until use for virus titration by TCID_50_, plaque-forming unit reduction assay, and real-time quantitative RT-PCR analysis. All assays were performed in triplicate.

### Post-treatment with recombinant Ad5 construct

The VerodogSLAM cells were seeded in 24-well plates (10^5^ cells/well in 500 μL DMEM + 10% FBS) and incubated for 24 h (37 °C, 5% CO_2_). Cells were first infected with CDV/LDM-BTU-2 at the MOI of 0.01. Two hours later, viral inoculum was removed, cells were washed twice with DMEM, and the medium was replaced by 2% FBS culture medium. At 12 h post-inoculation, cells were transduced with Ad5Ni(1–3) at MOI values of 5,10, 20, and 30. After a 1-h adsorption period at 37 °C, the inoculums were removed, cells were washed twice with DMEM, the medium was replaced by 2% FBS culture medium, and the plates were placed in the incubator. After 72 h, the plates were frozen/thawed once, and cell lysates plus supernatant were harvested and stored at − 80 °C until use for viral analysis and measurement procedures as described under 2.6. Mock infections and CDV infections (no Ad5 construct pre-infection) were included in the experiment; Ad5SCR was evaluated under the same conditions. All assays were performed in triplicate.

### Ad5-shRNA treatment at various time intervals

The VerodogSLAM cells were first infected with CDV/LDM-BTU-2 at the MOI of 0.01 and then transduced with Ad5Ni(1–3) vector at either MOI 2.5 or 5 at 2, 12, and 24 h post-infection. The experiments were done in triplicates and repeated independently. Plates were incubated at 37 °C for 72 h. All subsequent steps were performed as explained earlier under 2.7. Mock infection and non-treated CDV infection controls were set up in parallel. Virus reduction analysis read out was based on viral titer (TCID_50_/mL), RNA copy number, and inhibition percentage, according to the methods described above.

### Plaque-forming unit reduction assay for Ad5-expressed shRNA treatment

The gene suppressive activity of Ad5Ni(1–3) was determined by measuring the reduction in the number of CDV infectious plaques relative to the untreated control. Briefly, confluent monolayers of VerodogSLAM cells by 2 × 10^5^ were plated in each well of 24-well plates and incubated for 24 h. Samples of the above Ad5 construct treatments were added to the cells, and after 2 h of incubation, the inoculums were removed and the cells were overlaid with 2.5% carboxymethyl cellulose (CMC) with 10% FBS and incubated for 72 h. After that, cell monolayers were fixed and stained with crystal violet solution, and the plaque numbers were counted. The Ad5SCR treatment samples were also evaluated under the same conditions. The percentage of plaque reduction (PR %) compared to untreated infected cells was calculated using the following formula: PR (%) = (C - T) × 100/C, where C is the mean of the number of plaques from triplicate untreated control wells and T is the mean of the number of plaques from triplicate treated wells.

### Quantification of transcripts and viral genome

Total RNA was extracted from cell lysate plus supernatant (500 *μ*L), using Trizol reagent (Invitrogen) according the manufacturer’s instructions. The RNA was eluted in 20 μL and stored at − 80 °C. The concentration and quality of RNA were checked using a NanoDrop 2000 spectrophotometer (Thermo Fisher Scientific). For each real-time assay, 100 ng of RNA template were analyzed by RT-qPCR, following the protocols of the GoTaq 1-Step RT-qPCR System (Promega) (final volume 20 ul). Primers for the CDV N gene (Additional file 4) were designed based on previously published sequences [58, 59]. Selection of primers for the L protein-encoding gene was conducted by multiple sequence alignment using the MEGA software v. 6. A conserved region of the CDV L gene was selected for designing the primers with a product length of 136 bp (Additional file 4). Primers sets were synthesized by Integrated DNA Technologies (IDT). The RNA measurement by SYBR green incorporation was carried out in an Applied Biosystems 7500 Real-Time PCR system (Applied Biosystems), using the following thermal cycling profile: reverse transcription at 50 °C for 30 min, activation of Taq polymerase at 95 °C for 10 min and 40 cycles consisting of denaturation at 95 °C for 10 s, annealing at 60 °C for 30 s, and polymerization at 72 °C for 30 s. At the end of the amplification, a melt curve was generated from 70 to 95 °C, and fluorescence data were collected every 0.3 °C during melting. Real-time RT-PCR data were analyzed with the Applied Biosystems 7500 Software v. 2.0.6 (Applied Biosystems). The RNA transcript levels of the genes of interest in treated and control cells were determined by absolute quantification using the standard curve method. Briefly, a 287-bp amplified N gene fragment resulting from conventional PCR with P1 and P2 primers (Additional file 4), described by Frisk et al. [59], was cloned into the pGEM-T Easy (Promega). The pGEM-inserted N fragment contained the amplicon sequence chosen for real-time qRT-PCR. Moreover, another pGEM-T Easy ligation was made with a 136-bp PCR product amplified from the CDV L gene with L1 and L2 primers (Additional file 4). After confirming and linearizing both pGEM-T Easy constructs with Spel restriction enzyme (Promega), linearized plasmids were used as templates for in vitro transcription with the MEGAshortscript T7 Transcription Kit (Ambion) according to the manufacturer’s instructions. Turbo DNase-treated transcripts were ethanol-precipitated and resuspended in RNAse-free water. Synthetic RNAs were quantified using a Qubit 2.0 Fluorometer (Thermo Fischer Scientific), and the copy number was determined using the following formula: (X g/*μ*L DNA / [transcript length in base pairs × 340]) × 6.022 × 10^23^ = Y ssRNA molecules/*μ*L. Standard curve was constructed with five points in triplicate from serial 10-fold dilutions of transcripts with an amplification efficiency E = 98.5% (slope = − 3.286, R^2^ = 0.998). The RNA samples were tested in duplicate, and the inhibition of CDV replication was expressed as RNA copy number.

### Statistical analysis

Statistical analysis was performed by two-way ANOVA (analysis of variance) using the GraphPad Prism Software v. 5.01 for Windows (GraphPad Software, La Jolla, California, USA). The Tukey test was used for pairwise comparisons among means. Values of cell viability were calculated from a linear regression equation. All graphs were produced based on the means ± standard errors from three independent experiments. A *p*-value < 0.05 was considered statistically significant.

## Supplementary information

Additional file 1 Nucleotide sequences of designed shRNAs against CDV.

Additional file 2 Schematic representation of mRNA target sites, shRNA expression plasmids and predicted secondary structure of shRNAs. (A) Diagram of the full CDV genome. Highlighted CDV-specific genes are indicated the boxed sites of the shRNAs target sequences for nucleoprotein (Ni1, Ni2 and Ni3) and large polymerase mRNAs (Li1, Li2 and Li3). The shRNA control encodes a scrambled sequence. (B) The selected shRNA sequences were inserted into plasmid vectors under the control of human U6 RNA pol-lll.

Additional file 3 CPE scores were defined as (1) CPE < 25%, (2) CPE between 25 and 50%, (3) CPE between 51 and 75% and (4) CPE > 75%.

Additional file 4 Primers used in real-time and conventional RT-PCR assays for CDV.

Additional file 5 Control scrambled shRNA delivered using pENTR/U6-SCR displayed no meaningful suppressive activity against CDV N (A) and L (B) genes. RNA copy numbers were determined by real-time absolut quantification. (C) Transfection of scrambled shRNA did not affect viral replication. Virus titers were measured by TCID50 method. Values are the mean ± standard error from three independent experiments.

Additional file 6 Toxic effects induced by recombinant Ad5 viruses in VerodogSLAM cells. 24 h-plated cells were transduced with Ad5 constructs at various MOI values (10–200). Cell viability was measured by the MTT assay at 72 hpi. The dotted line in the graph indicates the corresponding cytotoxic concentration for 20% of cell culture (CC20). Each data point represents the mean ± standard error of three independent experiments done with eight replicates each.

## Data Availability

The datasets analyzed in this study are available from the corresponding author upon reasonable request.

## Abbreviations

CDV: *Canine distemper virus*
shRNA: short hairpin RNA
MeV: *Measles virus*
H: Hemagglutinin
M: Matrix protein
N: Nucleoprotein
L: Large polymerase
P: Phosphoprotein
RNAi: RNA interference
dsRNA: double-stranded RNA
PTGS: Post-transcriptional gene silencing
RISC: RNA-induced silencing complex
ssRNA: single-stranded RNA
PPRV: *Peste-des-petits-ruminants virus*
RPV: *Rinderpest virus*
CPE: Cytopathic effect
Ad5: Recombinant replication defective *human Adenovirus type 5* vector
BLD: Below limit of detection
