# Supplementary material for: Multi-targeted gene silencing strategies inhibit replication of Canine morbillivirus
Source: BMC Vet Res. 2020 Nov 19;16:448. doi: 10.1186/s12917-020-02671-2 (PMC7676405; doi:10.1186/s12917-020-02671-2)
Supplement: Supplementary file 1 — Nucleotide sequences of designed shRNAs against CDV. [file 12917_2020_2671_MOESM1_ESM.docx]

**Additional file 1.** Table**.** Nucleotide sequences of designed shRNAs against CDV.

| **Name** |  |  | **Target site^1^** | **Strand** | **Oligonucleotide sequence** |
| --- | --- | --- | --- | --- | --- |
| **Ni1** |  |  | N (1118-1138) | Top | 5’-*CACCG*GGAGTTGGTGTTGAACTTGAcgaaTCAAGTTCAACACCAACTCCC-3’ |
|  |  |  |  | Bottom | 5’-*AAAA*GGGAGTTGGTGTTGAACTTGAttcgTCAAGTTCAACACCAACTCC*C*-3’ |
| **Ni2** |  |  | N (648-668) | Top | 5’-*CACCG*CAGTGACTGCTCCTGATACTcgaaAGTATCAGGAGCAGTCACTGC-3’ |
|  |  |  |  | Bottom | 5’-*AAAA*GCAGTGACTGCTCCTGATACTttcgAGTATCAGGAGCAGTCACTG*C*-3’ |
| **Ni3** |  |  | N (1278-1298) | Top | 5’-*CACCG*CTCAGCTAGTGTCAGAAATAcgaaTATTTCTGACACTAGCTGAGC-3’ |
|  |  |  |  | Bottom | 5’-*AAAA*GCTCAGCTAGTGTCAGAAATAttcgTATTTCTGACACTAGCTGAG*C*-3’ |
|  |  |  |  |  |  |
| **Li1** |  |  | L (14602-14622) | Top | 5’-*CACCG*GAGTGTTGATTGTTACAAGTcgaaACTTGTAACAATCAACACTCC-3’ |
|  |  |  |  | Bottom | 5’-*AAAA*GGAGTGTTGATTGTTACAAGTttcgACTTGTAACAATCAACACTC*C*-3’ |
| **Li2** |  |  | L (14797-14817) | Top | 5’-*CACCG*CGACTGGGTTCAAGGATTTAcgaaTAAATCCTTGAACCCAGTCGC-3’ |
|  |  |  |  | Bottom | 5’-*AAAA*GCGACTGGGTTCAAGGATTTAttcgTAAATCCTTGAACCCAGTCG*C*-3’ |
| **Li3** |  |  | L (12705-12725) | Top | 5’-*CACCG*CATTCGTCAAATCTCCTAGTcgaaACTAGGAGATTTGACGAATGC-3’ |
|  |  |  |  | Bottom | 5’-*AAAA*GCATTCGTCAAATCTCCTAGTttcgACTAGGAGATTTGACGAATG*C*-3’ |
|  |  |  |  |  |  |
| **SCR** |  | No CDV target | | Top | 5’-*CACCG*CAACTATTGATCTCGCTAcgaaTAGCGAGATCAATAGTTGC-3’ |
|  |  |  |  | Bottom | 5’-*AAAA*GCAACTATTGATCTCGCTAttcgTAGCGAGATCAATAGTTG*C*-3’ |

Sense and antisense target sequences are underlined. Overhangs for directing the ds oligos ligation into the the pENTR/U6 RNAi entry vector were in italic type. Loop sequences were in lowercase word.

^1^This indicates the CDV gene and location of the 21 nts corresponding to the sense strand of the short hairpin construct-encoded siRNA, on the CDV genome; numbers indicated correspond to CDV isolate 164071 (Accession number EU716337.1).
