# Supplementary figures and images for: Multi-targeted gene silencing strategies inhibit replication of Canine morbillivirus
Source: BMC Vet Res. 2020 Nov 19;16:448. doi: 10.1186/s12917-020-02671-2 (PMC7676405; doi:10.1186/s12917-020-02671-2)

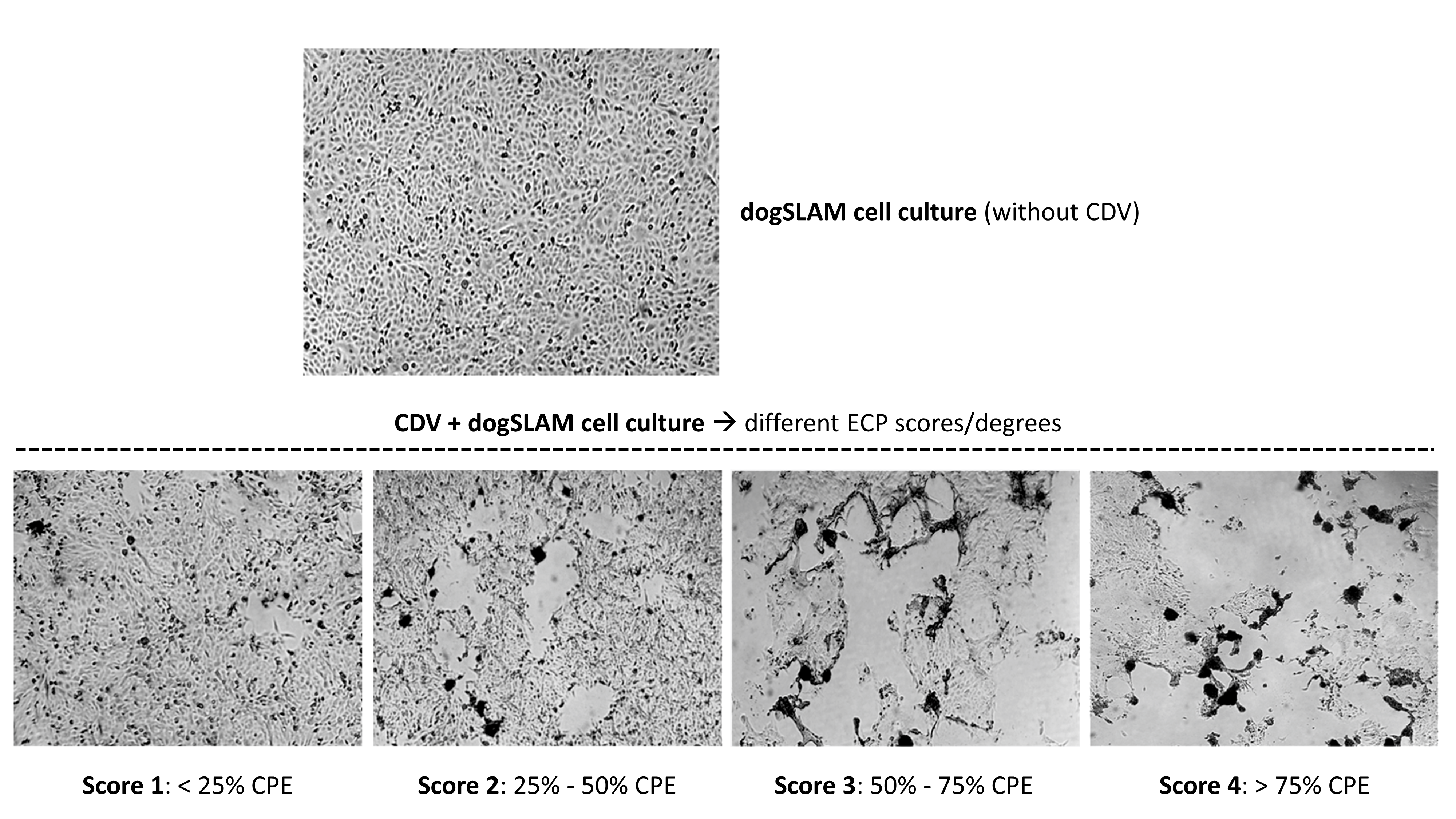

Supplement: Supplementary file 3 — CPE scores were defined as (1) CPE < 25%, (2) CPE between 25 and 50%, (3) CPE between 51 and 75% and (4) CPE > 75%. [file 12917_2020_2671_MOESM3_ESM.tif]

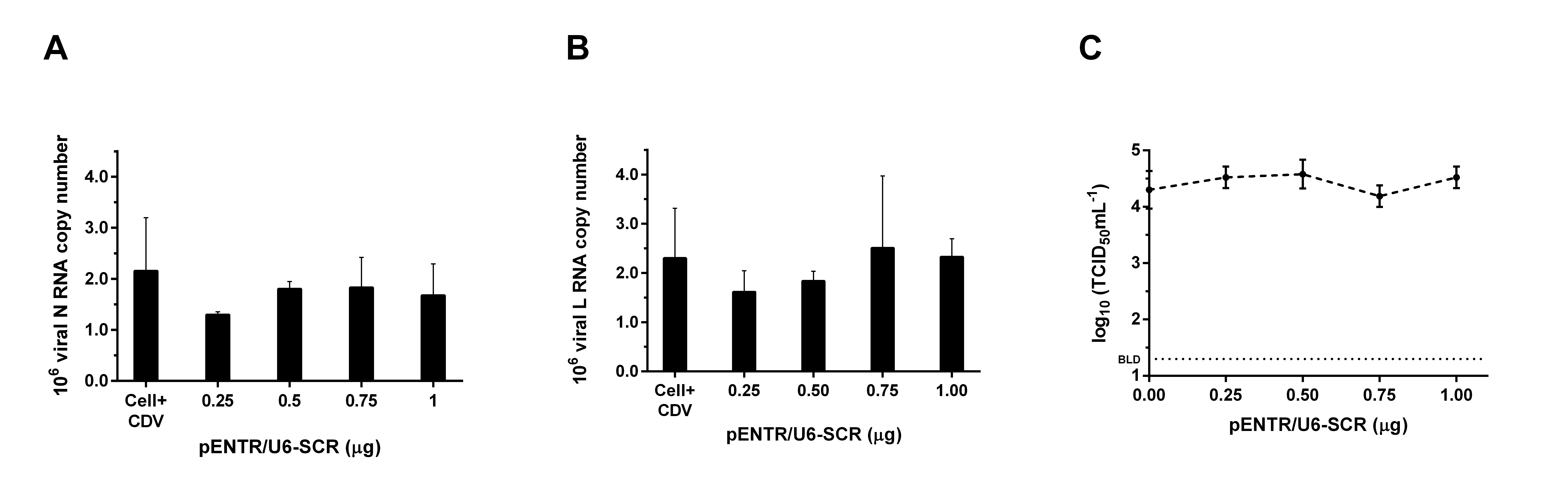

Supplement: Supplementary file 5 — Control scrambled shRNA delivered using pENTR/U6-SCR displayed no meaningful suppressive activity against CDV N (A) and L (B) genes. RNA copy numbers were determined by real-time absolut quantification. (C) Transfection of scrambled shRNA did not affect viral replication. Virus titers were measured by TCID50 method. Values are the mean ± standard error from three independent experiments. [file 12917_2020_2671_MOESM5_ESM.tif]

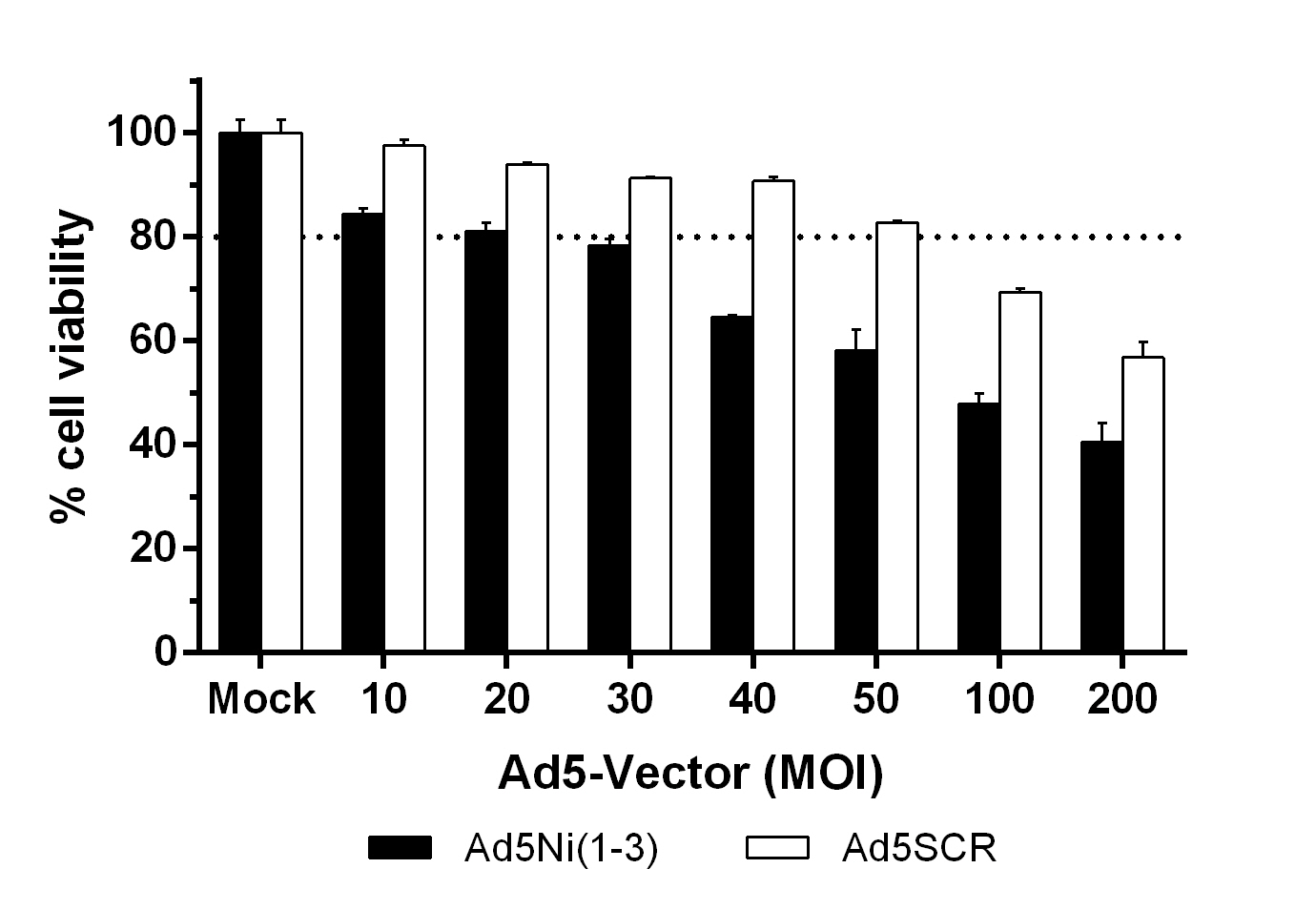

Supplement: Supplementary file 6 — Toxic effects induced by recombinant Ad5 viruses in VerodogSLAM cells. 24 h-plated cells were transduced with Ad5 constructs at various MOI values (10–200). Cell viability was measured by the MTT assay at 72 hpi. The dotted line in the graph indicates the corresponding cytotoxic concentration for 20% of cell culture (CC20). Each data point represents the mean ± standard error of three independent experiments done with eight replicates each. [file 12917_2020_2671_MOESM6_ESM.jpg]
