# Supplementary material for: Multi-targeted gene silencing strategies inhibit replication of Canine morbillivirus
Source: BMC Vet Res. 2020 Nov 19;16:448. doi: 10.1186/s12917-020-02671-2 (PMC7676405; doi:10.1186/s12917-020-02671-2)
Supplement: Supplementary file 4 — Primers used in real-time and conventional RT-PCR assays for CDV. [file 12917_2020_2671_MOESM4_ESM.docx]

**Additional file 4.** Table**.** Primers used in real-time and conventional RT-PCR assays for CDV.

| **Primer** | **CDV gene** | **Sequence (5’-3’)** | **Sense** | **Position** | **Product length** |
| --- | --- | --- | --- | --- | --- |
| **CDV-F^a^** | **N** | AGTTAGTTTCATCTTAACTATCAAATT | + | 905-931 | 87 bp |
| **CDV-R^a^** |  | TTAACTCTCCAGAAAACTCATGC | - | 966-987 |  |
| **L1^b^** | **L** | AGTGGCGACTGGGTTCAAG | + | 14793-14811 | 136 bp |
| **L2^b^** |  | GTCTCCCTGCTCTAAGACC | - | 14910-14928 |  |
| **P1^c^** | **N** | ACAGGATTGCTGAGGACCTAT | + | 769-789 | 287 bp |
| **P2^c^** |  | CAAGATAACCATGTACGGTGC | - | 1055-1035 |  |

^a^ qRT-PCR [44]

^b^ qRT-PCR. Design of primers based on CDV isolate 164071 (Accession number EU716337.1).

^c^ Conventional RT-PCR [45].
